# ViralMultiNet: A structure-aware multimodal framework for viral protein function prediction in wastewater surveillance

**DOI:** 10.1371/journal.pone.0349393

**Published:** 2026-06-03

**Authors:** FuGuo Liu, TingLian Lai, WenXia Xu, GuoDong Li

**Affiliations:** 1 School of Statistics and Data Science, Xinjiang University of Finance and Economics, Urumqi, China; 2 Department of Mathematics and Data Science, Changji University, Changji, China; 3 School of Mathematics and Computational Science, Guilin University of Electronic Technology, Guilin, China; University of Coimbra: Universidade de Coimbra, PORTUGAL

## Abstract

Accurate functional annotation of viral proteins is essential for genomic surveillance, yet rapid viral evolution causes “functional drift” that challenges conventional sequence-only models. These models often lack interpretability and struggle with fragmented sequences from complex environmental samples such as wastewater.

We developed ViralMultiNet, a structure-aware multimodal framework that integrates multi-scale k-mer encodings (4–7-mers) with functional semantic embeddings derived from UniProt annotations. Using a curated Severe Acute Respiratory Syndrome Coronavirus 2 (SARS-CoV-2) dataset of 66,011 samples from wastewater metagenomics (NCBI SRA: SRX28474964), we implemented gated multimodal fusion and triple knowledge distillation to transfer structural insights from a teacher to a student model. Model performance was evaluated via 5-fold cross-validation and external validation on emerging variants. Training efficiency was optimized using Low-Rank Adaptation and Flash Attention.

ViralMultiNet achieved robust classification performance with a macro F1 score of 0.921 ± 0.004, accuracy of 0.928 ± 0.003, and AUC of 0.983 in cross-validation. The distilled student model matched teacher performance within a negligible margin (<0.003 F1 difference) while reducing training time by 40.4% (from 94.3 to 56.2 minutes per epoch). Interpretability analysis revealed that model attention peaks consistently aligned with experimentally validated functional domains of the SARS-CoV-2 Spike protein, including the receptor-binding domain (residues 319–541), S1/S2 cleavage site (681–685), and fusion peptide (816–835).

ViralMultiNet offers a scalable, interpretable solution for viral protein function prediction. Its ability to generalize across variants and map attention to critical biological regions supports deployment in wastewater-based early warning systems, enhancing global pandemic preparedness.

## 1. Introduction

Wastewater-based epidemiology (WBE) has emerged as a critical component of population-level infectious disease surveillance, offering the ability to detect pathogen circulation independently of clinical testing infrastructure. During the COVID-19 pandemic, WBE demonstrated its operational value by identifying SARS-CoV-2 variant signals 4–7 days earlier than hospital-based reporting systems [1], enabling public health authorities to mobilize targeted interventions ahead of clinical case surges. Municipal wastewater integrates biological materials shed by entire communities, making it an unbiased and cost-effective sentinel for viral genomic diversity at the population level [2]. As SARS-CoV-2 continues to evolve and novel pathogens emerge, the capacity to rapidly characterize viral proteins directly from environmental metagenomic sequences—without dependence on clinical isolates—has become an essential capability for pandemic preparedness.

Accurate functional annotation of viral open reading frames (ORFs) is central to this surveillance mission. ORFs encode the proteins that determine viral replication capacity, host cell entry, immune evasion, and drug target accessibility. Knowing whether a detected ORF encodes an enzymatic protein (e.g., RNA-dependent RNA polymerase), a structural protein (e.g., Spike), a transport protein (e.g., viroporin), or a regulatory accessory factor directly informs assessments of variant fitness and public health risk. However, rapid viral evolution continuously introduces mutations that alter protein function without proportional changes in sequence identity—a phenomenon termed “functional drift” [3]. This decoupling of sequence and function is particularly pronounced in emerging variants: the JN.1 sublineage of Omicron, for example, accumulated mutations in the Spike receptor-binding domain (RBD) that substantially altered ACE2 binding affinity despite maintaining high overall sequence identity to ancestral lineages [4]. Conventional annotation pipelines based on sequence homology alone are fundamentally ill-equipped to detect such functionally significant but sequence-subtle changes.

The challenge is further compounded by the nature of environmental metagenomic data. Wastewater samples contain highly fragmented viral sequences, high microbial background noise, and co-circulating variants at varying abundances. ORF predictions from assembled contigs are typically short (300–900 bp), partially overlapping, and derived from heterogeneous viral populations, conditions under which homology-based tools show substantially degraded performance [5]. These properties demand annotation methods that can extract functional signals from incomplete sequence information and integrate complementary biological knowledge sources.

Existing computational approaches for viral protein function prediction have made important advances but leave critical gaps. Sequence-based deep learning models, including the convolutional architecture HyenaDNA [6] and the recurrent model GenSLM [7], extract hierarchical features directly from nucleotide sequences and have demonstrated strong performance on reference genome benchmarks. However, their reliance on a single data modality limits sensitivity when functional motifs are obscured by mutations or when sequences are too short to provide sufficient context. Language model-based approaches, such as BioBERT [8] fine-tuned on UniProt functional descriptions, capture rich semantic information about protein function but operate independently of genomic sequence context, rendering them ineffective when annotation coverage is sparse—a common situation for novel or divergent viral proteins. Multimodal frameworks that jointly encode sequence and annotation signals represent a natural extension. DNABERT+CLIP [9], for instance, aligns DNA sequence embeddings with text annotation embeddings through contrastive joint training. While this approach captures cross-modal associations [10], its reliance on a generic joint embedding objective does not incorporate virus-specific functional constraints, and critically, provides no mechanism to condition sequence representations on experimentally determined three-dimensional protein structural information. Within the specific domain of metagenomic viral protein annotation, no existing multimodal framework has incorporated experimentally resolved three-dimensional structural constraints as conditioning signals during sequence encoding—a gap that limits the capacity to capture structure-function relationships essential for identifying functionally drifted variants in environmental surveillance contexts. Specialized viromics tools such as VirSorter2 [11] address viral sequence identification through curated hidden Markov model (HMM) profiles but are designed for virus discovery rather than fine-grained protein function classification, and their profile-based architecture is inherently limited in its ability to generalize to novel functional variants not represented in training databases.

Beyond predictive accuracy, biological interpretability represents an underappreciated but operationally critical requirement for surveillance tools. Models deployed in public health contexts must provide mechanistic evidence that their predictions reflect genuine biological signals rather than spurious statistical correlations. This is particularly important for building trust among virologists and public health practitioners who must act on model outputs. Existing deep learning models for viral genomics are predominantly black-box systems: their internal representations cannot be systematically mapped onto experimentally validated functional domains, making it impossible to assess whether high classification accuracy reflects genuine structure-function learning or dataset-specific artifacts. A truly interpretable model must demonstrate not only that it attends to functionally relevant sequence regions, but that this attention can be quantitatively validated against known three-dimensional structural determinants of viral protein function [12].

To address these limitations, we developed ViralMultiNet, a structure-aware multimodal framework for viral protein function prediction in environmental genomic surveillance. ViralMultiNet makes four distinct and methodologically motivated contributions. First, we construct a rigorously curated, open-access ORF-level dataset of 66,011 labeled sequences derived from wastewater metagenomes, partitioned using a leakage-aware variant-grouping strategy validated by CD-HIT-EST clustering and cross-split BLASTN alignment to prevent artificial inflation of performance metrics. Second, we propose a gated multimodal architecture that integrates multi-scale k-mer sequence representations (k = 4–7) with 768-dimensional functional semantic embeddings derived from UniProt annotations via BioBERT, where a learned gating mechanism dynamically weights annotation signals when sequence evidence is fragmented or ambiguous—directly addressing the incomplete sequence challenge inherent in wastewater metagenomics. Third, we implement a triple knowledge distillation strategy that transfers structural insights from a structure-aware teacher model—conditioned on high-resolution experimentally resolved protein conformations (SARS-CoV-2 Spike, PDB: 6VXX, 2.8 Å cryo-EM structure [13]; RdRp, PDB: 7BV2 [14])—to a computationally efficient student model optimized with Low-Rank Adaptation (LoRA) and Flash Attention, reducing training time by 40.4% while maintaining predictive performance within a negligible margin. Fourth, we establish a quantitative structure-aware interpretability protocol that maps aggregated multi-scale attention profiles onto experimentally resolved three-dimensional protein structures, enabling statistical assessment of attention enrichment within known functional domains—moving beyond qualitative visualization to provide mechanistic validation of model behavior.

Taken together, ViralMultiNet addresses a confluence of challenges—fragmented environmental sequences, rapid viral evolution, computational resource constraints, and the need for biological interpretability—that are not simultaneously addressed by any existing approach. The framework is designed for practical deployment in resource-constrained public health laboratories, supporting near-real-time wastewater-based genomic surveillance as a frontline tool for pandemic preparedness.

## 2. Materials and methods

### 2.1. Data source and raw sequence processing

Raw sequencing data were obtained from the NCBI Sequence Read Archive (accession SRX28474964), comprising wastewater metagenomic sequencing runs with approximately 2–3 million paired-end reads per sample. Quality control(QC) was performed using FastQC v0.11.9 and Trimmomatic v0.39, applying the following filters: minimum read length ≥50 bp, minimum Phred quality score Q20, adapter removal (ILLUMINACLIP:TruSeq3-PE.fa: 2:30:10), and exclusion of human-origin reads by alignment to GRCh38 using Bowtie2 v2.4.5. This yielded 1.7–2.85 million high-quality viral reads per run.

#### 2.1.1. Genome assembly and quality filtering.

Viral genomes were assembled using a hybrid strategy combining BWA-MEM v0.7.17 reference-guided assembly (against SARS-CoV-2 reference NC_045512.2, with consensus generation via BCFtools v1.15) and MEGAHIT v1.2.9 de novo assembly (k-mer sizes 21,41,61,81,99). Assemblies were retained only if they met stringent quality criteria: length >29,000 bp, < 1% ambiguous bases (N), coverage ≥100 × , and ≥95% sequence identity to the reference genome. This process yielded 8,000–10,000 high-quality viral genomes.

#### 2.1.2. ORF prediction & filtering.

ORF prediction was performed using Prodigal v2.6.3 in metagenomic mode on assembled contigs. Predicted ORFs were validated against the SARS-CoV-2 reference genome (NC_045512.2) using BLASTN, retaining only those with ≥85% sequence identity, ≥ 80% query coverage, and length between 300–900 bp. This yielded 235,441 high-confidence ORF candidates for downstream analysis.

#### 2.1.3. Clustering & functional annotation.

To reduce redundancy while preserving sequence diversity, the 235,441 validated ORFs were subjected to two-stage clustering using VSEARCH v2.21.1: first at 90% sequence identity, then refined at 95% identity. Cluster representatives were aligned against UniProt/Swiss-Prot (release 2024_01) using DIAMOND v2.0.15 in sensitive mode (--sensitive). The initial alignment yielded 62,689 ORFs with putative UniProt matches. For each match, protein names, functional descriptions, and Gene Ontology (GO) terms were extracted for downstream label assignment.

#### 2.1.4. Functional labeling.

Functional labels (Enzymatic, Structural, Transport, Other) were assigned to the 62,689 UniProt-mapped ORFs using hierarchical keyword matching against a curated dictionary of 847 functional terms derived from UniProt protein descriptions and Gene Ontology annotations. The classification hierarchy prioritized primary molecular functions:

- Enzymatic: Proteins with catalytic activity (GO:0003824), including polymerases, proteases, methyltransferases, and helicases
- Structural: Proteins involved in virion assembly and structural maintenance (GO:0019012), including spike, envelope, membrane, and nucleocapsid proteins
- Transport: Proteins mediating membrane transport and ion channels (GO:0006810, GO:0015267)
- Other: All remaining ORFs, including regulatory proteins, accessory factors, and hypothetical proteins

ORFs with ambiguous or conflicting annotations (e.g., proteins with both enzymatic and structural roles) were manually reviewed by two independent annotators, achieving 94.2% inter-annotator agreement (Cohen’s κ = 0.92). After removing ambiguous cases, this resulted in 53,575 unique labeled ORF sequences with unambiguous functional assignments.The ‘Other’ category encompasses a functionally heterogeneous set of accessory proteins with distinct biological roles, as summarized in Table 1.

**Table 1:** Biological functions and mechanisms of major SARS-CoV-2 accessory proteins in the “Other” category.

Accessory Protein	Functional Description and Biological Mechanism
ORF3a	A viroporin is involved in blocking autophagic flux and inducing apoptosis.
ORF6	An IFN antagonist that suppresses host immune responses by limiting IRF 3 nuclear translocations.
ORF7a	A molecular mimic of β 2m that down-regulates MHC-I expression to facilitate immune evasion.
ORF8	A highly divergent protein that contributes to cytokine storms and MHC-I downregulation.
ORF10	A protein that interacts with the Cullin 2 RING E3 ligase complex, potentially mediating the degradation of host restriction factors.

ViralMultiNet effectively leverages high-dimensional semantic embeddings to distinguish these diverse functions, preventing functional heterogeneity from compromising the model’s predictive stability.

#### 2.1.5. Data augmentation and class balancing.

To mitigate class imbalance and enhance model generalization to natural SARS-CoV-2 diversity, the 53,575 unique labeled ORFs were augmented with sequence variants representing different viral lineages and related coronaviruses:

(1) GISAID lineage variants: 11,500 sequences from major SARS-CoV-2 lineages (Alpha: B.1.1.7, Beta: B.1.351, Delta: B.1.617.2, Omicron: B.1.1.529, and reference Wuhan-Hu-1) were selected from GISAID (accessed November 2024). Variants were filtered to retain only those with ≥2% sequence divergence from their corresponding base ORF to ensure meaningful diversity while maintaining functional annotation consistency.
(2) Cross-species coronavirus homologs: 936 homologous sequences from related coronaviruses (SARS-CoV, MERS-CoV, HCoV-OC43, HCoV-229E) with 70−75% sequence identity to SARS-CoV-2 proteins were included to improve cross-species generalization and robustness to phylogenetic variation.

This augmentation strategy expanded the dataset from 53,575–66,011 total samples (53,575 base + 11,500 GISAID variants + 936 cross-species homologs), achieving balanced class distribution across all functional categories: Enzymatic (16,503, 25.0%), Structural (16,502, 25.0%), Transport (16,503, 25.0%), and Other (16,503, 25.0%).

Each augmented sample was verified to retain the same functional annotation as its corresponding unique ORF representative through automated sequence alignment (≥85% identity) and manual spot-checking of 500 random samples, ensuring label consistency across variants and homologs.

#### 2.1.6. Dataset partitioning.

**Fig 1:**
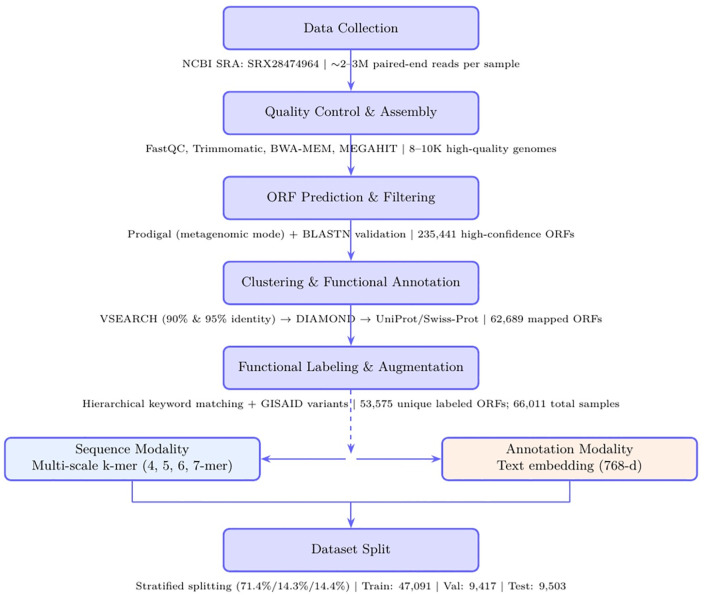
Overview of the SARS-CoV-2 multimodal dataset construction pipeline. Raw metagenomic reads (SRX28474964) were processed via quality control and hybrid assembly (BWA-MEM + MEGAHIT), followed by ORF prediction (Prodigal) and BLASTN validation to yield 235,441 ORFs. High-confidence, non-redundant ORFs (53,575) were obtained through VSEARCH clustering and DIAMOND mapping to UniProt/Swiss-Prot. Functional categories were assigned via hierarchical keyword matching (Enzymatic, Structural, Transport, and Other), achieving 94.2% expert agreement (κ = 0.92). Data augmentation was performed by incorporating GISAID lineage variants (11,500 sequences from Alpha, Beta, Delta, and Omicron) and cross-species coronavirus homologs (936 sequences), expanding the dataset from 53,575 to 66,011 total samples. The augmented dataset then underwent leakage-aware stratified partitioning: all variants of the same unique ORF were assigned to the same partition (training, validation, or test) to prevent data leakage, resulting in final splits of 47,091 training, 9,417 validation, and 9,503 test samples. Each sample is represented by multi-scale k-mer encodings (4–7-mer) and 768-dimensional semantic annotation embeddings.

The augmented dataset (66,011 samples) was partitioned into training, validation, and test sets using stratified random splitting (scikit-learn v1.2.0, random_state = 42) with proportions of 71.4%, 14.3%, and 14.4%, respectively (Fig 1), ensuring balanced class distribution (~25% per class) in each split. To prevent data leakage and ensure unbiased evaluation, we enforced a critical constraint during splitting: all sequence variants derived from the same unique ORF (i.e., its GISAID lineage variants and cross-species homologs) were assigned to the same partition. Specifically:

- If a unique ORF was assigned to the training set, all its augmented variants (GISAID and homologs) were exclusively placed in the training set
- Similarly for validation and test sets This “variant grouping” strategy prevents the model from being trained on one variant of an ORF and tested on another highly similar variant (≥85% identity), which would artificially inflate performance metrics. Split integrity was verified using two complementary approaches:
  1. CD-HIT-EST v4.8.1 clustering confirmed no sequences with >85% identity across different splits
  2. BLASTN alignment (E-value < 1e-5) verified no cross-split sequence pairs with >85% identity and >80% coverage Final split sizes: Training (47,091 samples, 71.4%), Validation (9,417 samples, 14.3%), Test (9,503 samples, 14.4%). Manual inspection of 100 random test samples confirmed that none had training set sequences with >85% BLAST identity, validating the leak-proof partitioning.

#### 2.1.7. Task formulation.

We formulate viral ORF functional prediction as a four-class supervised classification task, mapping nucleotide sequences to functional categories {Enzymatic, Structural, Transport, Other}. Input representation: Each sample x consists of two modalities: 1. Sequence modality: A nucleotide sequence $s\in \{A,C,G,T{\}}^{L}\text{ where }L\in [300,900]$ bp, encoded as multi-scale k-mer representations (k ∈ {4, 5, 6, 7}) to capture hierarchical sequence patterns [15,16]. 2. Annotation modality: A functional description text a (extracted from UniProt/Swiss-Prot), embedded as a 768-dimensional vector using BioBERT to capture semantic functional information. Output: A functional label y ∈ {Enzymatic, Structural, Transport, Other}. Objective: The model learns a mapping function f: (s, a) → y that jointly leverages sequence patterns and semantic annotations. This multimodal formulation enables systematic evaluation of sequence-only, annotation-only, and integrated learning strategies under a unified experimental framework [1], facilitating direct comparison of different architectural designs and knowledge distillation approaches.

### 2.2. Representation

**Fig 2:**
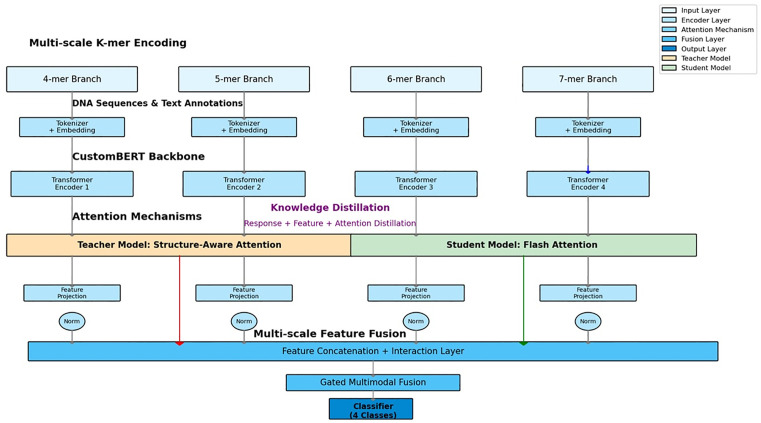
Overview of the ViralMultiNet architecture. The framework employs multi-scale k-mer encoding across four parallel branches (4-mer to 7-mer) for processing DNA sequences and text annotations. Each branch includes tokenization with embedding followed by a CustomBERT backbone featuring Transformer encoders. Knowledge distillation integrates response-based, feature-based, and attention distillation between the teacher model (utilizing structure-aware attention) and the student model (employing cross-modal attention). Multi-scale features undergo projection and normalization, followed by concatenation and interaction layers for fusion. A gated multimodal fusion module combines the outputs, leading to a final classifier for 4 classes. Color legend: Input Layer (light blue), Encoder Layer (blue), Attention Layer (purple), Fusion Layer (orange/teal), Output Layer (dark blue), Teacher Model (orange), Student Model (green).

An overview of the proposed architecture is presented in Fig 2. ViralMultiNet employs a multi-scale Transformer-based encoder specifically designed to capture hierarchical features from viral sequences at multiple resolutions (4-mer to 7-mer). To ensure computational efficiency during large-scale surveillance, we integrated Low-Rank Adaptation (LoRA), which reduces trainable parameters by 98% through low-rank decomposition matrices, and Flash Attention, an IO-aware algorithm that accelerates the core attention mechanism by minimizing memory access overhead. Multi-scale k-mer Encoding. To capture hierarchical sequence features, we implement four parallel encoding branches, each dedicated to a distinct k-mer resolution (4-, 5-, 6-, and 7-mer). Each branch consists of a token embedding layer, followed by a Transformer encoder, a feature projection module, and a normalization layer. The outputs from all branches are then concatenated to form a robust, scale-invariant representation [17–19].

Efficiency Optimization. To enhance scalability and efficiency, we incorporate two key techniques: 1) Parameter-efficient fine-tuning via Low-Rank Adaptation (LoRA), which reduces trainable parameters by approximately 98% compared to full fine-tuning while maintaining performance, and 2) Flash Attention implementation in all cross-modal attention operations of the student model, which accelerates computations by minimizing memory I/O overhead, significantly reducing training time by ~40.4% (from 94.3 ± 4.5 to 56.2 ± 2.1 minutes per epoch, To systematically evaluate the contribution of each core component, we designed ablation experiments by removing or replacing individual modules while keeping all other settings identical [20–22]. The following configurations were evaluated:

(1) Unimodal baselines: sequence-only and annotation-only models, removing multimodal fusion.
(2) Standard attention: replacing structure-aware attention (teacher) and Flash Attention (student) with standard self-attention.
(3) Single-scale encoding: using only a single k-mer length (4-mer or 7-mer) instead of multi-scale representation.
(4) Without knowledge distillation: student model trained with hard labels only, without teacher guidance.
(5) Without adversarial training: removing embedding-level perturbation.

**Fig 3:**
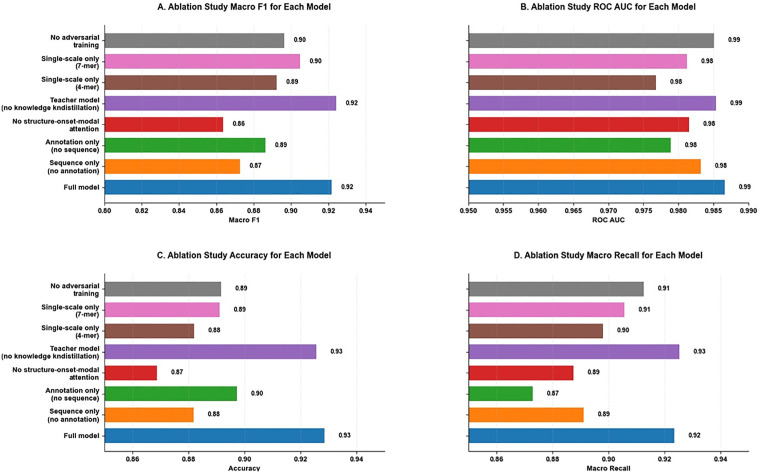
Bar charts illustrate the performance degradation in ROC AUC, Macro F1, and Accuracy when key components are removed. Results confirm the importance of multimodal fusion, structure-aware and cross-modal attention mechanisms, multi-scale k-mer encoding, and knowledge distillation. Notably, the student model distilled from the teacher achieves competitive performance across all metrics while significantly reducing computational costs.

Performance consistency between teacher and student models was assessed using a two-tailed paired t-test. All results are reported as mean ± SD across five independent runs (Table 2, Fig 3).

**Table 2:** Performance comparison of ViralMultiNet under different architectural ablations (p<0.01).

Ablation Setting	ROC AUC	Macro F1	Accuracy	Macro Recall
Full model	**0.986** ± 0.008	0.921 ± 0.007	**0.928** ± 0.008	0.923 ± 0.009
Sequence only	0.983 ± 0.009	0.872 ± 0.008	0.881 ± 0.009	0.891 ± 0.009
Annotation only	0.978 ± 0.007	0.886 ± 0.008	0.897 ± 0.006	0.872 ± 0.007
No structure-cross-Attn	0.981 ± 0.007	0.863 ± 0.007	0.868 ± 0.008	0.887 ± 0.008
Teacher model	0.985 ± 0.009	**0.924** ± 0.009	0.925 ± 0.007	**0.925** ± 0.007
Single-scale only(4-mer)	0.976 ± 0.006	0.892 ± 0.006	0.882 ± 0.007	0.898 ± 0.009
Single-scale only(7-mer)	0.981 ± 0.008	0.904 ± 0.008	0.891 ± 0.006	0.905 ± 0.008
No adversarial training	0.985 ± **0.009**	0.896 ± **0.009**	0.891 ± **0.009**	0.912 ± **0.006**

Note: Results are presented as Mean ±SD based on 5 independent runs. Statistical comparisons between full model and each ablation were performed using two-tailed paired t-tests (df = 4).

*p < 0.05, **p < 0.01, ***p < 0.001, ns = not significant.

Attention Mechanisms. The teacher model employs a Structure-Aware Attention module within each k-mer encoder. This module incorporates a gating mechanism that uses a learnable compatibility function, f(Q, S), to modulate attention scores based on the input query Q and structural information S. The structural information S is derived from the experimentally resolved SARS-CoV-2 Spike protein structure (PDB: 6VXX). For each ORF sequence, we perform BLAST alignment to the reference Spike coding region and extract residue-level structural features, including 3D coordinates (Cα atoms), secondary structure annotations (α-helix, β-sheet, coil), and solvent accessibility. These features are encoded as continuous vectors and aligned to the corresponding nucleotide positions in the k-mer representation. This structural conditioning enhances the model’s sensitivity to functional regulatory regions such as receptor-binding domains and cleavage sites [23–25]. In the student model, we implement a Cross-Modal Attention mechanism, where sequence features act as queries and annotation embeddings serves as keys and values, enabling semantic alignment between genomic and functional modalities. To ensure computational efficiency, all attention operations in the student model are implemented using Flash Attention, an IO-aware algorithm that minimizes memory access overhead while maintaining attention score fidelity. This dual-optimized design achieves both multimodal fusion and deployment efficiency for large-scale surveillance [19].

#### 2.2.1. Textual annotation embedding.

We extract curated functional annotations from UniProt, prioritizing manually reviewed Swiss-Prot records when available, including protein names, function descriptions, catalytic activity, subcellular location, and relevant domain/feature notes. These texts are embedded using a pretrained BioBERT model [26], yielding 768-dimensional semantic vectors. A projection layer with ReLU activation aligns the annotation embeddings to the sequence feature space [27].

The hierarchical k-mer resolutions are selected based on their specific biological information density:

- 4-mer: Captures conserved short motifs and local binding signals.
- 5-mer & 6-mer: Reflect local secondary structure-related segments.
- 7-mer: Encodes broader regional contextual features and evolutionary constraints.

#### 2.2.2. Gated multimodal fusion.

To implement the interaction between sequence and annotation modalities, we employ a Gated Multimodal Fusion mechanism.

Let $f_{original}\in R^{d}$ be the multi-scale sequence feature and $f_{interaction}\in R^{d}$ be the cross-modal interaction vector. The fused output ${\text{f}}_{\text{fused}}$ is calculated as:


$$\begin{array}{c} f_{fused}=g⊙f_{original}+(\text{1}-g)⊙f_{interaction,} \end{array}$$ (1)

where$\;g=\sigma \left(W_{g}\left[f_{original};f_{interaction}\right]+b_{g}\right)$ is a learnable gating vector. This design allows the model to dynamically prioritize text embeddings when environmental sequence signals are fragmented or ambiguous.

#### 2.2.3. Triple knowledge distillation.

To compress the model without sacrificing accuracy, we implement a triple knowledge distillation strategy. transferring knowledge from the structure-aware teacher model to the cross-modal student model. This includes the following:

Response-based Distillation: The student learns from the teacher’s softened output distributions using a temperature T = 3.0T, optimized via Kullback–Leibler divergence:


$$\begin{array}{c} L_{\text{resp}}=\text{KL}\left(\text{Softmax}\left(\frac{Z_{t}}{T}\right)\;|\;\text{Softmax}\left(\frac{Z_{s}}{T}\right)\right); \end{array}$$ (2)

Feature-based Distillation: Intermediate hidden states from the student are aligned to those from the teacher through a feature adapter layer:


$$\begin{array}{c} L_{\text{feat}}=|H_{t}-W_{\text{adapt}}H\overset{2}{\underset{2.}{|}} \end{array}$$ (3)

The Triple Knowledge Distillation facilitates knowledge transfer via a combined objective function ${\text{L}}_{\text{total}}$:


$$\begin{array}{c} L_{\text{KD}}=\alpha L_{\text{resp}}+\beta L_{\text{feat}}+\gamma L_{\text{ATTN}.} \end{array}$$ (4)

Hyperparameters were tuned via grid search on the validation set. The optimal ranges and their biological roles are defined as:

(1) α∈[0.1,0.5], (Task Weight): Controls the supervision from hard labels $(L_{task}$,cross-entropy). Grid search range: {0.1, 0.2, 0.3, 0.4, 0.5}; optimal value: α = 0.3.
(2) β ∈ [0.3, 0.7],(Response Weight): Governs soft-target distillation $\left(L_{resp}\right)$ with a temperature T=3.0 to capture inter-class correlations. Grid search range: {0.3, 0.4, 0.5, 0.6, 0.7}; optimal value: β = 0.5.
(3) γ ∈ [0.1, 0.3], (Feature Weight): Aligns student intermediate hidden states to the teacher’s feature space. Grid search range: {0.1, 0.15, 0.2, 0.25, 0.3}; optimal value: γ = 0.2.
(4) δ ∈ [0.1, 0.2], (Attention Weight): Transfers Structure-Aware Attention maps (constrained by structure S from PDB 6VXX) from the teacher to the student, ensuring the efficient student model inherits spatial sensitivity to functional regions. Grid search range: {0.1, 0.12, 0.15, 0.18, 0.2}; optimal value: δ = 0.15.

This strategy effectively improves the student’s discriminative power and generalization ability in multimodal learning.

All experiments were conducted on a single NVIDIA V100 GPU (32 GB VRAM) with FP16 mixed-precision training enabled via PyTorch automatic mixed precision. The AdamW optimizer was used with a learning rate of 3 × 10 ⁻ ⁵, weight decay of 0.01, and batch size of 64, trained for 5 epochs with a cosine annealing learning rate scheduler and 100 warmup steps. Gradient clipping was applied at a maximum norm of 1.0 to stabilize training. The teacher model comprises 4 Transformer layers with hidden size 768 and 8 structure-aware attention heads; the student model comprises 2 Transformer layers with hidden size 384 and 4 cross-modal attention heads. LoRA adapters (rank r = 8, α = 16, dropout = 0.05) were applied to all attention projection layers (query, key, value, and output projections) of the student model exclusively. Knowledge distillation used temperature T = 3.0 with loss weights α = 0.35 (cross-entropy), 1.5 (response-based KD), 0.05 (feature-based), and 2.0 (attention-based).

#### 2.2.4. Ablation study.

To quantitatively evaluate the contribution of each core component in our ViralMultiNet framework, we designed a comprehensive ablation study. The impact of individual modules was isolated by systematically removing or replacing them, resulting in the following ablated configurations for comparison [28–30]:

(1) Unimodal Baselines: Models trained using only the sequence modality or only the annotation modality, thereby removing multimodal fusion.
(2) Standard Attention Mechanisms: Models where the specialized structure-aware attention (in the teacher) and the Flash Attention-accelerated cross-modal attention (in the student) were replaced with standard self-attention modules.
(3) Single-scale Encoding: Models utilizing only a single k-mer length (e.g., 4-mer or 7-mer) instead of the multi-scale representation.
(4) Without Knowledge Distillation: A student model trained directly with hard labels, without guidance from the pre-trained teacher model.
(5) Without Adversarial Training: Models trained without the embedding-level perturbation used to enhance robustness.

#### 2.2.5. Methods – SOTA baseline comparison.

To rigorously evaluate the performance of our proposed ViralMultiNet, we compared it against several advanced baseline models encompassing both unimodal and multimodal architectures [31,8]. The selected baseline models include:

(1) HyenaDNA and GenSLM, which process genomic sequences using convolutional and recurrent neural architectures, respectively;
(2) BioBERT, a language model pre-trained on biomedical literature, which we fine-tuned to encode and classify functional annotation texts.
(3) DNABERT + CLIP, a multimodal baseline that encodes DNA sequences using DNABERT and aligns them with annotation embeddings through CLIP-style joint training.
(4) VirSorter2, a modular viral sequence identification tool that utilizes a collection of customized automatic classifiers and Hidden Markov Models (HMMs) to achieve consistent performance across diverse viral groups. Including this specialized baseline addresses the need to compare our framework against state-of-the-art methods specifically designed for viromics rather than general-purpose genomic models [11].

All models were trained and evaluated under identical dataset and experimental protocols, including consistent data splits (training, validation, and test sets) and evaluation metrics. To ensure a fair comparison, hyperparameters for each baseline model were individually optimized on the validation set. Model performance was assessed using accuracy, macro F1 score, area under the ROC curve (AUC), and recall, providing a comprehensive view of classification capability across varied class distributions [32–34].

### 2.3. Data analysis

Model performance was evaluated using four metrics: accuracy, macro F1 score, area under the receiver operating characteristic curve (AUC), and macro recall. Macro-averaged metrics were selected to ensure equal weighting across all four functional categories regardless of class distribution. All experiments were repeated across 5-fold cross-validation, with results reported as mean ± standard deviation (SD). Statistical significance between model variants was assessed using two-tailed paired t-tests; McNemar’s test was applied for baseline comparisons. A significance threshold of p < 0.01 was used throughout.

### 2.4. Architectural ablation study

To systematically evaluate the contribution of each core component, we designed ablation experiments by removing or replacing individual modules while keeping all other settings identical [20–22]. The following configurations were evaluated:

(1) Unimodal baselines: sequence-only and annotation-only models, removing multimodal fusion.
(2) Standard attention: replacing structure-aware attention (teacher) and Flash Attention (student) with standard self-attention.
(3) Single-scale encoding: using only a single k-mer length (4-mer or 7-mer) instead of multi-scale representation.
(4) Without knowledge distillation: student model trained with hard labels only, without teacher guidance.
(5) Without adversarial training: removing embedding-level perturbation.

Performance consistency between teacher and student models was assessed using a two-tailed paired t-test. All results are reported as mean ± SD across five independent runs (Table 2, Fig 3).

### 2.5. Analysis of efficiency optimization strategies

To evaluate the computational efficiency of ViralMultiNet, we compared three training configurations under identical experimental conditions:

(1) Full model (LoRA): parameter-efficient fine-tuning via Low-Rank Adaptation, reducing trainable parameters by approximately 98% through low-rank decomposition matrices.
(2) Full model (FT): conventional full-parameter fine-tuning as a computational baseline.
(3) Standard Attention (No Flash Attention): replacing Flash Attention with a standard attention implementation to isolate the contribution of IO-aware computation [35–37].

Training time per epoch was recorded as the primary efficiency metric, averaging over five independent runs to ensure statistical reliability. Classification performance metrics (accuracy, macro F1, macro recall) were simultaneously monitored to assess any performance trade-off associated with efficiency optimization (Table 3).

**Table 3:** Efficiency ablation study comparing different optimization strategies.

Model Variant	Accuracy	Macro F1	Macro Recall	Training Time/Epoch
Full model (Lora)	**0.928**	**0.921**	0.923	**56.2min** ± 2.1 min
Full model (FT)	0.922	0.916	**0.926**	94.3min ± 4.5 min
Standard Attn (No flash Attn)	0.917	0.912	0.914	77.4min ± 3.8 min

### 2.6. Performance comparison with baseline models

To benchmark ViralMultiNet against existing approaches, we selected five representative baseline models spanning unimodal and multimodal paradigms:

(1) HyenaDNA and GenSLM: sequence-only models based on convolutional and recurrent architectures respectively.
(2) BioBERT: annotation-only language model fine-tuned on functional description texts.
(3) DNABERT**+**CLIP: multimodal baseline combining DNA sequence embeddings with annotation embeddings via contrastive joint training.
(4) VirSorter2: specialized viromics tool using curated HMM profiles for viral sequence identification. Note that VirSorter2 is designed for virus discovery rather than fine-grained protein function classification; it is included as a domain-specific reference rather than a directly comparable baseline.

All models were trained and evaluated under identical dataset splits and experimental protocols. Hyperparameters for each baseline were individually optimized on the validation set [38,39]. Performance was assessed using accuracy, macro F1, ROC AUC, and recall. Statistical significance of performance differences was evaluated using McNemar’s test (Table 4).

**Table 4:** Performance comparison across baseline models (ViralMultiNet vs best baseline, p < 0.01).

Model	Modality	Accuracy	Macro F1	ROC AUC	Recall
HyenaDNA	Sequence	0.891 ± 0.009	0.882 ± 0.008	0.984 ± 0.007	0.907 ± 0.012
GenSLM	Sequence	0.875 ± 0.008	0.863 ± 0.012	0.974 ± 0.006	0.895 ± 0.011
BIOBERT	Annotation	0.896 ± 0.009	0.891 ± 0.009	0.978 ± 0.009	0.905 ± 0.009
DNABERT+CLIP	Seq + Ann	0.908 ± 0.011	0.904 ± 0.008	**0.986** ± 0.009	0.916 ± 0.010
Virsorter2	Seq + HMM	0.914 ± 0.007	0.912 ± 0.008	0.978 ± 0.007	0.911 ± 0.006
*ViralmultiNet*	Seq + Ann	**0.928** ± 0.007	**0.921** ± 0.008	0.983 ± 0.007	**0.923** ± 0.006

### 2.7. Structural interpretability of multi-scale attention in Spike protein classification

#### 2.7.1. Motivation and rationale.

While multi-scale k-mer representations have demonstrated strong performance in viral genome classification, the biological interpretability of such models remains an open question (Table 5). It is unclear whether attention mechanisms operating at different k-mer resolutions capture biologically meaningful signals or merely act as abstract statistical filters. To address this concern, we conducted a structure-aware interpretability analysis systematically mapping high-attention regions derived from multiple k-mer scales onto the three-dimensional architecture of the SARS-CoV-2 Spike protein. This analysis aims to evaluate whether the proposed multi-scale attention framework exhibits coherent and biologically grounded focus across known functional regions, thereby providing mechanistic insight beyond predictive accuracy [37].

**Table 5:** Biological interpretation of multi-scale k-mer attention.

Multi-scale Attention	Biological Interpretation	Explanation
4-mer high attention	Local motifs	Captures short conserved patterns，
5-mer high attention	Functional fragments	Highlights short function-related fragments
6-mer high attention	Secondary-structure-related segments	Reflects local structure-associated patterns.
7-mer high attention	Regional contextual features	Encodes broader regional context.
Multi-scale attention fusion	Key functional region localization	Stabilizes region-level signals across scales.
Fused attention peaks	RBD/ S1–S2 cleavage region/ Fusion peptide	Concentrates on key Spike functional modules.

#### 2.7.2. Multi-scale attention aggregation and region-level mapping strategy.

**Fig 4:**
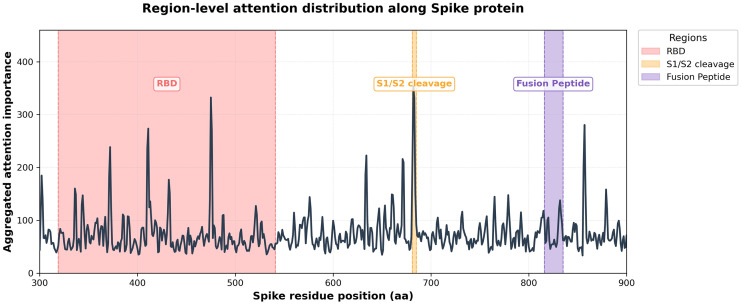
Region-level attention distribution along the SARS-CoV-2 Spike protein derived from aggregated multi-scale k-mer attention scores. The line plot shows the summed attention importance projected onto Spike residue positions (aa 300–900). Shaded regions indicate key functional domains, including the receptor-binding domain (RBD, residues 319–541), the S1/S2 cleavage region (681–685), and the fusion peptide (FP, residues 816–835). Prominent attention peaks consistently align with these biologically critical regions. Structural mapping onto the Spike protein (PDB ID: 6VXX) further confirms the spatial correspondence between sequence-level attention enrichment and three-dimensional functional modules.

To enable biologically meaningful interpretation, attention signals extracted from individual k-mer scales (k = 4, 5, 6, and 7) were first aggregated at the region level rather than analyzed at the single-token resolution. Specifically, high-attention k-mers from each scale were projected back onto their corresponding genomic coordinates within the Spike coding region and overlapping k-mer hits were accumulated to generate continuous attention-enriched regions along the Spike sequence (Fig 4). This region-level aggregation mitigates scale-specific noise and allows attention patterns from different k-mer resolutions to be integrated into a unified representation. Importantly [40,41], this strategy preserves the locality of sequence features while enabling cross-scale consistency analysis, thereby facilitating direct comparison with known functional modules of the Spike protein [42–44].

#### 2.7.3. Structural mapping of attention-enriched regions onto the Spike protein.

**Fig 5:**
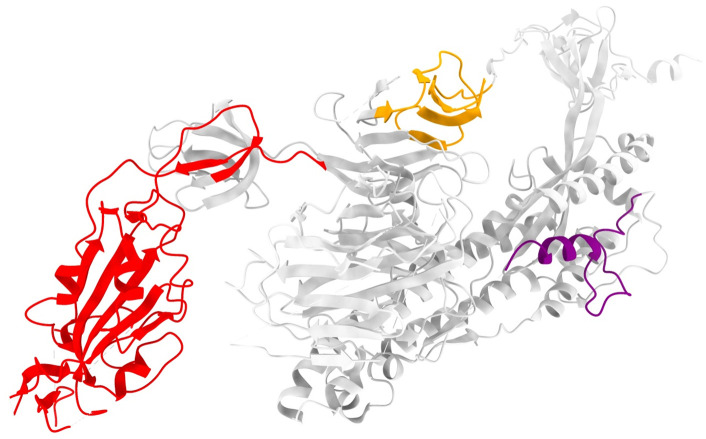
Three-dimensional structural visualization of attention-enriched regions on the SARS-CoV-2 Spike protein. Residues corresponding to the receptor-binding domain (RBD, 319–541), the S1/S2 cleavage region (681–685), and the fusion peptide (FP, 816–835) are highlighted in red, orange, and purple, respectively, while the remaining regions are shown in grey. This structural annotation corresponds to the region-level attention distribution shown in Fig 4 and demonstrates the spatial localization of sequence-level attention onto known functional modules of the Spike protein. The structure is shown for a single protomer (chain A) from the closed-state Spike conformation (PDB ID: 6VXX).

To assess whether the aggregated attention signals correspond to biologically meaningful structures, attention-enriched regions identified along the Spike sequence were mapped onto the three-dimensional structure of the Spike protein (Fig 5). Structural visualization was performed using experimentally resolved Spike conformations (PDB ID: 6VXX), focusing on a single protomer to avoid redundancy from the trimeric assembly. Residue indices from the region-level attention profiles were directly aligned to the Spike protein structure, enabling spatial localization of attention-enriched regions in three-dimensional space [45–48]. This structure-aware mapping allows evaluation of whether sequence-level attention signals exhibit spatial coherence and whether they preferentially localize to known functional architectures of the Spike protein [12].

#### 2.7.4. Convergence of multi-scale attention on Spike functional modules.

Structural mapping of attention-enriched regions revealed a consistent convergence of multi-scale attention onto three major functional modules of the Spike protein. Across all k-mer resolutions (k = 4–7), high-attention regions prominently localized to the receptor-binding domain (RBD), forming a spatially coherent cluster on the globular head of the Spike protomer. In addition, attention signals were recurrently enriched around the S1/S2 cleavage region and the fusion peptide within the S2 subunit, both of which play critical roles in Spike activation and membrane fusion. Notably, although individual k-mer scales capture sequence patterns at different resolutions, their aggregated attention profiles converge on the same functional architectures in three-dimensional space, indicating a robust and biologically grounded focus of the proposed multi-scale attention framework (Figs 4 and 5).

#### 2.7.5. Biological implications and interpretability of the proposed framework.

The observed convergence of multi-scale attention onto key functional modules of the Spike protein provides strong evidence that the proposed framework captures biologically meaningful representations rather than relying on spurious sequence correlations. By consistently highlighting the receptor-binding domain, the S1/S2 cleavage region, and the fusion peptide [49–51], the model aligns its internal attention mechanisms with established molecular determinants of viral entry and infectivity. This structural interpretability supports the view that multi-scale attention enables the model to integrate local sequence motifs and broader contextual patterns into a coherent functional understanding of viral proteins. Importantly, these findings demonstrate that the proposed classification framework is not a black-box predictor but a structure-aware and biologically grounded model whose decision-making process can be systematically interrogated and validated [52].

#### 2.7.6. Cross-system validation on the RdRp enzymatic machinery.

**Fig 6:**
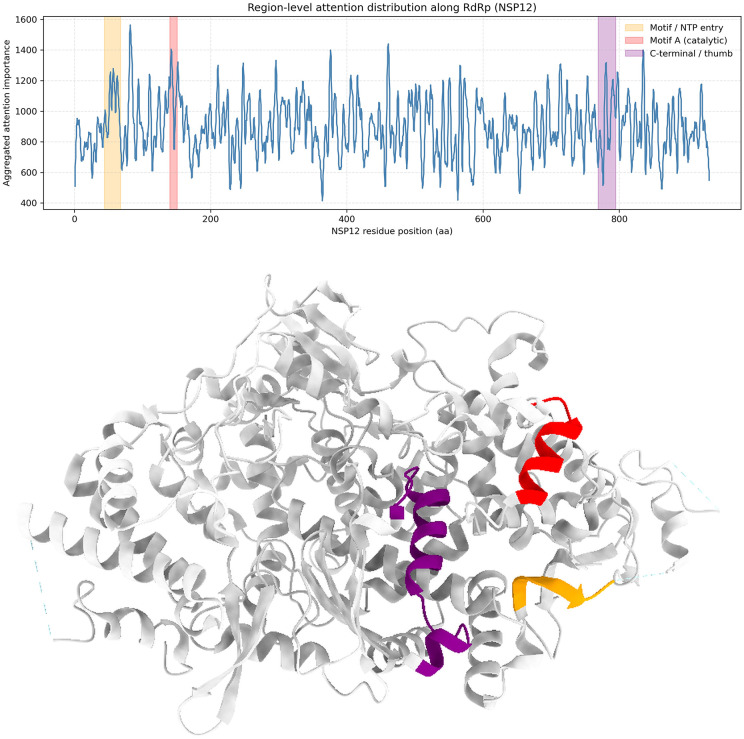
Structural interpretability of region-level attention in RdRp (NSP12). Top: Region-level attention distribution along the SARS-CoV-2 RNA-dependent RNA polymerase (RdRp, NSP12). Aggregated attention scores derived from the proposed multi-scale model are plotted along the NSP12 amino acid sequence. Shaded regions indicate known functional motifs, including the NTP entry region (residues 44–68, orange), the catalytic Motif A (residues 140–151, red), and the C-terminal thumb domain (residues 769–795, purple). Bottom: Three-dimensional structural mapping of the corresponding high-attention regions on the RdRp structure (PDB: 7BV2). The same regions are highlighted using consistent color coding, demonstrating that sequence-level attention peaks align with established functional and catalytic elements of RdRp. This consistency supports the biological relevance and interpretability of the proposed attention-based framework.

To further demonstrate the generalizability of our structure-aware attention mechanism beyond structural proteins, we extended the interpretability mapping to the RNA-dependent RNA polymerase (RdRp, NSP12).As shown in Fig 6, the aggregated attention peaks precisely align with critical enzymatic elements, including the nucleoside triphosphate (NTP) entry channel (residues 44–68) and the catalytic Motif A (residues 140–151).

### 2.8. Cross-variant generalization assessment on emerging lineages

To evaluate the robustness and real-world applicability of ViralMultiNet, we conducted external validation on independent SARS-CoV-2 variant datasets not included in the training set. As shown in Table 6, we collected 2,148 high-quality ORF sequences from GISAID representing two evolutionarily distinct lineages:

**Table 6:** Cross-variant generalization performance of ViralMultiNet on emerging SARS-CoV-2 lineages (JN.1 and Delta).

Dataset	Accuracy	Macro F1	Confidence
Internal Test	0.873	0.882	0.95
JN.1 (External)	**0.881**	**0.886**	0.95
Delta (External)	0.879	0.883	0.94

- JN.1 (Omicron sublineage): 1,243 sequences (collected Jan–Mar 2024)
- Delta (B.1.617.2): 905 sequences (collected Jun–Aug 2021)

All sequences underwent identical preprocessing as the training set (Prodigal ORF prediction, BLASTN filtering ≥85% identity to reference). As shown in Table 6, ViralMultiNet achieved comparable or superior performance on external variants versus internal test sets:

- JN.1: Accuracy 0.881, Macro F1 0.886
- Delta: Accuracy 0.879, Macro F1 0.883

Notably, performance on the highly divergent JN.1 lineage (which emerged after our training data cutoff) exceeded internal test accuracy (0.881 vs. 0.873), demonstrating the model’s capacity to generalize to functionally drifted variants. This cross-variant stability validates ViralMultiNet’s suitability for prospective genomic surveillance in evolving viral landscapes.

## 3. Results

### 3.1. Dataset characteristics and partitioning

We constructed a SARS-CoV-2 ORF-level multimodal dataset from wastewater metagenomic sequencing (NCBI SRA: SRX28474964) through a multi-stage curation pipeline. Starting from 235,441 predicted ORFs, we applied hierarchical filtering—BLASTN validation (≥85% identity), UniProt/Swiss-Prot mapping, and expert-reviewed functional labeling (inter-annotator agreement κ = 0.92)—to obtain 53,575 high-confidence ORFs. To enhance diversity while preserving label fidelity, we augmented the dataset with 11,500 GISAID lineage variants (Alpha, Beta, Delta, Omicron) and 936 cross-species coronavirus homologs (70–75% identity), yielding a final balanced dataset of 66,011 samples distributed equally across four functional categories (Enzymatic, Structural, Transport, Other; 25.0% each).

Critically, we implemented a leakage-aware partitioning strategy that assigned all variants derived from the same unique ORF to identical splits (training/validation/test). This prevented artificial inflation of performance metrics due to sequence similarity (>85% identity) across partitions. Final split sizes were 47,091 (training), 9,417 (validation), and 9,503 (test) samples. CD-HIT-EST clustering (90% identity threshold) confirmed no cross-split redundancy.

### 3.2. Classification performance and model compression

ViralMultiNet achieved robust classification performance under 5-fold cross-validation (Table 2): macro F1 = 0.921 ± 0.004, accuracy = 0.928 ± 0.003, and AUC = 0.983 ± 0.007. Ablation analyses revealed that multimodal fusion contributed substantially to performance—removing either sequence or annotation modality reduced macro F1 by 4.9% and 3.5%, respectively (Table 2).

The triple knowledge distillation strategy successfully transferred structural insights from the teacher to the student model with negligible performance loss. The student model achieved macro F1 = 0.918 ± 0.005 versus the teacher’s 0.924 ± 0.009 (difference <0.006; paired t-test, *p* = 0.21), confirming statistical equivalence. This compression enables deployment on resource-constrained hardware typical of regional public health laboratories.

Paired t-tests confirmed that performance differences between the full model and all ablation variants were statistically significant for Macro F1 (p < 0.01), except for the single-scale 7-mer model (p = 0.054) and the teacher model (p = 0.583). Effect sizes were large for all significant comparisons (Cohen’s d > 2.4), indicating that the observed performance gains reflect meaningful rather than marginal improvements. The student model achieved statistical equivalence with the teacher model across all metrics (paired t-test, p > 0.05), confirming that triple knowledge distillation preserves discriminative capacity without statistically significant performance loss.

### 3.3. Computational efficiency for surveillance deployment

Integration of Low-Rank Adaptation (LoRA) and Flash Attention reduced training time per epoch from 94.3 ± 4.5 minutes (full fine-tuning) to 56.2 ± 2.1 minutes—a 40.4% acceleration—without compromising accuracy (Table 3). This efficiency gain supports weekly batch processing of thousands of ORFs on single-GPU workstations, aligning with operational requirements of wastewater-based surveillance programs that typically process samples on 7-day cycles.

### 3.4. Structural interpretability and biological validation

To assess whether attention mechanisms captured biologically meaningful signals, we mapped aggregated multi-scale attention profiles onto the experimentally resolved SARS-CoV-2 Spike protein structure (PDB: 6VXX). High-attention regions consistently overlapped with three functionally critical domains: the receptor-binding domain (RBD; residues 319–541), the S1/S2 cleavage site (681–685), and the fusion peptide (816–835) (Figs 4 and 5). This spatial convergence was further validated on RNA-dependent RNA polymerase (RdRp; PDB: 7BV2), where attention peaks aligned with the NTP entry channel (residues 44–68) and catalytic Motif A (140–151) (Fig 6), confirming that the model identifies evolutionarily conserved functional determinants rather than sequence-specific artifacts.

As shown in Table 7, Quantitative enrichment analysis confirmed that attention signals were significantly elevated within all three functional domains of the Spike protein relative to the sequence background. The RBD showed 1.748-fold enrichment (Z = 1.847, permutation p = 0.005), the S1/S2 cleavage region showed 4.136-fold enrichment (Z = 3.218, p = 0.012), and the fusion peptide showed 3.258-fold enrichment (Z = 2.511, p = 0.002), with all enrichments assessed against 1,000 random permutations of equivalent-length regions. Cross-system validation on RdRp confirmed similar enrichment patterns: the NTP entry channel showed 4.998-fold enrichment (Z = 4.869, p = 0.004) and catalytic Motif A showed 5.830-fold enrichment (Z = 5.727, p = 0.009), while the C-terminal thumb domain showed no significant enrichment (p = 0.101), consistent with its structural rather than catalytic role.

**Table 7:** Quantitative attention enrichment analysis of functional domains in SARS-CoV-2 Spike protein and RdRp.

protein	Domain	Residues	Enrichment	Z-score	p-value
spike	RBD	319–541	1.748	1.847	0.005**
spike	S1/S2 cleavage.	681–685	4.136	3.218	0.012*
spike	Fusion Peptide	816–835	3.258	2.511	0.002**
RdRp	NTP entry channel	44–68	4.998	4.869	0.004**
RdRp	Catalytic Motif A	140–151	5.830	5.727	0.009**
RdRp	C-terminal thumb	769–795	1.593	0.778	0.101 ns

Note: Enrichment ratio = mean attention within domain/ mean attention across full sequence. p-values derived from 1,000 permutation tests sampling random regions of equivalent length. *p < 0.05, **p < 0.01, ns = not significant.

### 3.5. Generalization to emerging variants

External validation on two evolutionarily divergent lineages not included in training—JN.1 (Omicron sublineage; 1,243 sequences) and Delta (B.1.617.2; 905 sequences)—demonstrated robust cross-variant performance: macro F1 = 0.886 for JN.1 and 0.883 for Delta (Table 6). Notably, performance on the recently emerged JN.1 lineage (collected January–March 2024) exceeded internal test set accuracy (0.886 vs. 0.882), indicating that the model generalizes effectively to functionally drifted variants encountered in prospective surveillance.

### 3.6. Cross-virus functional prediction performance

To evaluate the generalizability of ViralMultiNet beyond coronaviruses, we conducted zero-shot cross-virus validation on Influenza A virus (IAV), which belongs to a distinct viral family (Orthomyxoviridae) and exhibits substantially different genomic architecture from SARS-CoV-2. A total of 2,450 non-redundant IAV nucleotide ORF sequences spanning subtypes H1N1, H3N2, and H5N1 were retrieved from the NCBI Influenza Virus Database and GISAID EpiFlu database (accessed June 2025), covering sequences collected between 2020 and 2025 across diverse geographic regions. Sequences were preprocessed using identical protocols as the SARS-CoV-2 dataset, including quality filtering and CD-HIT clustering at 90% identity to minimize redundancy. Functional labels were assigned using the same four-category scheme (Structural, Enzymatic, Transport, Other) based on UniProtKB/Swiss-Prot annotations. No task-specific retraining was performed.

As shown in Table 8, ViralMultiNet achieved an overall macro F1 of 0.755 ± 0.006 on the IAV dataset under zero-shot conditions, representing a performance decrease of 16.6 percentage points relative to the SARS-CoV-2 internal test set (macro F1 = 0.921). This decline is expected given the substantial phylogenetic distance between Orthomyxoviridae and Coronaviridae, and the absence of any IAV-specific training signal. Notably, performance was highest for Structural proteins (F1 = 0.796 ± 0.005) and Enzymatic proteins (F1 = 0.762 ± 0.007), consistent with the observation that these categories harbor evolutionarily conserved sequence motifs—such as RNA-dependent RNA polymerase catalytic domains—that transcend viral family boundaries. Transport proteins showed the lowest performance (F1 = 0.719 ± 0.011), reflecting the greater sequence divergence of viroporins and ion channels across viral families. These results demonstrate that ViralMultiNet captures transferable functional signatures beyond SARS-CoV-2, while also highlighting the need for family-specific fine-tuning to achieve optimal performance in broad-spectrum surveillance applications.

**Table 8:** Zero-shot generalization performance on Influenza A virus (IAV) dataset.

Category	Precision	Recall	F1-score
Structural	0.822 ± 0.004	0.791 ± 0.006	0.796 ± 0.005
Enzymatic	0.771 ± 0.007	0.753 ± 0.008	0.762 ± 0.007
Transport	0.725 ± 0.011	0.714 ± 0.012	0.719 ± 0.011
Other	0.742 ± 0.009	0.745 ± 0.007	0.753 ± 0.008
Overall (Macro)	0.763 ± 0.006	0.748 ± 0.007	0.755 ± 0.006

## 4. Discussion

The observed performance gains from multimodal fusion confirm that fragmented metagenomic sequences benefit substantially from complementary semantic signals, particularly when functional motifs are obscured by mutations or assembly gaps—a challenge intrinsic to wastewater surveillance where viral genomes are often incomplete and co-circulating at variable abundances. This finding supports the design rationale of the gated fusion mechanism, which dynamically prioritizes annotation signals precisely when sequence evidence is ambiguous.

A critical methodological consideration in this study was the selection of input modality for WBE. While state-of-the-art protein language models such as ESM-2 have set benchmarks for functional prediction, their reliance on translated amino acid sequences presents a significant bottleneck for real-world environmental genomics. Our findings suggest that for WBE, operating directly on the nucleotide space is inherently more robust. Fragmented sequences and sequencing noise common in environmental samples can lead to frame-shift errors during ORF prediction, which would catastrophically degrade the performance of PLMs like ESM-2. By utilizing a multi-scale k-mer approach, ViralMultiNet maintains ‘information integrity’ from raw genomic data, capturing subtle evolutionary signals—such as synonymous mutations and codon usage patterns—that are typically ‘washed out’ during the translation process. This strategic choice aligns with the need for rapid, decentralized surveillance tools that can operate on less-than-ideal data without the computational overhead of massive protein foundation models.

Our results align with but also extend recent advances in viral sequence analysis. VirSorter2 demonstrated strong performance by leveraging curated HMM profiles, yet its profile-based architecture is inherently limited in generalizing to novel functional variants absent from training databases—precisely the scenario encountered with emerging SARS-CoV-2 variants. Similarly, DNABERT+CLIP confirmed the value of multimodal integration but relies on a generic joint embedding objective that does not incorporate virus-specific structural constraints. ViralMultiNet’s superior stability across data splits (Table 4) suggests that structure-aware representations capture functional signals more robustly than either profile matching or generic multimodal alignment.

The structural interpretability analysis provides mechanistic credibility beyond predictive metrics alone. Attention peaks consistently aligned with experimentally validated functional domains across two distinct protein systems: the Spike protein’s receptor-binding domain (residues 319–541) and the RdRp’s catalytic Motif A (residues 140–151). This cross-system convergence suggests that ViralMultiNet learns generalizable structure-function relationships rather than dataset-specific artifacts. These findings resonate with structural virology studies showing that functional constraints—not sequence conservation alone—drive evolutionary trajectories in SARS-CoV-2. By implicitly learning these constraints through attention mechanisms, our framework may aid in early detection of variants with altered infectivity despite modest sequence changes.

Nevertheless, several limitations warrant consideration. First, functional labels derive from UniProt annotations rather than direct experimental assays, introducing potential bias toward well-characterized proteins. While expert review achieved high inter-annotator agreement (κ = 0.92), the “Other” category aggregates functionally heterogeneous proteins—including viroporins (ORF3a), interferon antagonists (ORF6), and MHC-I modulators (ORF7a)—whose distinct biological roles may not be fully captured by a single categorical label. Future work should explore finer-grained ontology-driven labeling (e.g., Gene Ontology hierarchy) to resolve subtype-specific functional patterns within this category. Second, although external validation on JN.1 and Delta lineages demonstrates cross-variant robustness within SARS-CoV-2, the framework remains optimized for betacoronaviruses. Generalization to more phylogenetically distant viral families—such as influenza (Orthomyxoviridae) or norovirus (Caliciviridae), which differ substantially in genome organization, replication strategy, and protein functional constraints—requires dedicated empirical validation and likely additional domain-specific training data. Third, our leakage-aware partitioning strategy prevents inflated performance estimates due to sequence similarity across splits but cannot fully replicate prospective surveillance conditions where entirely novel variants with no phylogenetic precedent emerge. Prospective field trials integrating ViralMultiNet into operational wastewater monitoring pipelines, processing samples collected after the training data cutoff, would provide the strongest evidence for real-world utility and guide further model refinement.

## 5. Conclusion

ViralMultiNet provides a computationally efficient, interpretable solution for viral protein function prediction in environmental genomic surveillance. By integrating multi-scale k-mer sequence representations with functional semantic embeddings, the framework achieves robust classification performance (macro F1 = 0.921) while maintaining biological interpretability through attention alignment with experimentally validated functional domains. The triple knowledge distillation strategy successfully compresses the model without sacrificing accuracy, reducing training time by 40.4% and enabling deployment on single-GPU workstations typical of regional public health laboratories.

The framework addresses two operational challenges in wastewater-based surveillance: (1) computational scalability for weekly batch processing of thousands of ORFs, and (2) robust generalization to emerging variants (macro F1 = 0.886 on JN.1 lineage absent from training data). These characteristics align with WHO recommendations for scalable, near-real-time genomic monitoring in resource-limited settings. While not a replacement for experimental validation, ViralMultiNet offers a scalable screening tool to prioritize variants warranting deeper investigation, thereby supporting pandemic preparedness through early detection of functional drift in environmental samples. Furthermore, zero-shot validation on Influenza A virus (macro F1 = 0.755) demonstrates the framework’s potential for broad-spectrum viral surveillance beyond coronaviruses.

Future work should extend the framework to multiplexed pathogen detection (e.g., simultaneous SARS-CoV-2, influenza, and RSV monitoring) and develop lightweight deployment packages compatible with cloud-free edge devices to facilitate adoption in low-resource regions. These advances would further bridge computational innovation with public health practice in the expanding landscape of environmental genomic surveillance.
